# Changes in the negative logarithm of end-tidal hydrogen partial pressure indicate the variation of electrode potential in healthy Japanese subjects

**DOI:** 10.1038/s41598-023-42651-8

**Published:** 2023-09-19

**Authors:** Teruo Kiyama, Akira Tokunaga, Abumrad Naji, Adrian Barbul

**Affiliations:** 1 Department of Gastrointestinal and Hepato-Biliary-Pancreatic Surgery, Nippon Medical School, Bunkyo, Tokyo Japan; 2 Department of Surgery, TMG Asaka Medical Center, Asaka, Saitama Japan; 3 Department of Surgery, Vanderbilt University School of Medicine, Nashville, TN USA; 4 Present Address: Department of Surgery, Musashino Tokushukai Hospital, Nishi-Tokyo, Tokyo Japan

**Keywords:** Biochemistry, Biophysics, Physiology

## Abstract

Molecular hydrogen (H_2_) is produced by human colon microbiomes and exhaled. End-tidal H_2_ sampling is a simple method of measuring alveolar H_2_. The logarithm of the hydrogen ion (H^+^)/H_2_ ratio suggests the electrode potential in the solution according to the Nernst equation. As pH is defined as the negative logarithm of the H^+^ concentration, pH_2_ is defined as the negative logarithm of the H_2_ effective pressure in this study. We investigated whether changes in pH_2_ indicated the variation of electrode potential in the solution and whether changes in end-tidal pH_2_ could be measured using a portable breath H_2_ sensor. Changes in the electrode potential were proportional to (${\mathrm{pH}}_{2}-2\times \mathrm{pH}$) in phosphate-buffered solution (pH = 7.1). End-tidal H_2_ was measured in the morning (baseline) and at noon (after daily activities) in 149 healthy Japanese subjects using a handheld H_2_ sensor. The median pH_2_ at the baseline was 4.89, and it increased by 0.15 after daily activities. The variation of electrode potential was obtained by multiplying the pH_2_ difference, which suggested approximately + 4.6 mV oxidation after daily activities. These data suggested that changes in end-tidal pH_2_ indicate the variation of electrode potential during daily activities in healthy human subjects.

## Introduction

Molecular hydrogen (H_2_) is available in trace amounts in most ecosystems through atmospheric, biological, geochemical, and anthropogenic sources, and the atmosphere is critical for life as the main source of oxygen (O_2_) for aerobic respiration^1^. An obligately aerobic bacterium activates fermentative H_2_ production to survive reductive stress during hypoxia^2^. However, mammalian cells lack functional hydrogenase genes. H_2_ metabolism in the human gut microbiome is driven by fermentative H_2_ production and interspecies H_2_ transfer^3^. In 2007, it was reported that 2–4% H_2_ gas inhalation relieved brain ischemia–reperfusion injury in rats by selectively removing cytotoxic oxygen radicals^4^. H_2_ released from intestinal bacteria can suppress inflammation induced in the liver by concanavalin A^5^. H_2_ fermented by human microbiomes may affect aerobic metabolism under oxidative stress.

Stable aerobic and anaerobic states coexist in the lungs and colon of the human body. Intestinal gas contains up to 50% H_2_ gas. Mucus H_2_ concentrations were measured to be about 168 µM in the small intestines and 43 µM in the stomachs of live mice^6^. In general, biologically important gases behave like ideal gases; diffusion depends on the partial pressure gradient, but the dissolved gas concentration at equilibrium depends on both partial pressure and solubility. Although the alimentary tract is considered outside of the body, some of the H_2_ generated in the colon is partly absorbed, passes in the circulating blood to the lungs, and diffuses into the alveolar space, where its presence can be easily determined^7^. However, after gas-exchange in the lungs, H_2_ remains in arterial blood as well as the alveolar space according to Henry’s law and distributes throughout tissues until saturation^8,9^.

In plastic surgery, the H_2_ clearance technique was introduced for monitoring postoperative blood flow after free-tissue transfer in a clinical study^10^. Briefly, H_2_ is inhaled for 10–15 s and reaches the body tissue through arterial blood flow. Platinum electrodes are located in the tissue of interest. The generated electric current is proportional to the H_2_ partial pressure in the tissue^11^. The decay of H_2_ concentration (clearance effect) is registered by the electrode. The mathematical analysis of clearance curves allows quantitative expression of blood flow value. In gastroenterology, an increase in the rate of breath H_2_ excretion was introduced into clinical practice for the assessment of carbohydrate malabsorption and a simple method was used for measuring H_2_ concentration in alveolar air by end expiratory sampling in the 1970s^12,13^. The H_2_ breath test has been widely used for diagnostic purposes in adults and children^14,15^. Recently, an additional measurement of CH_4_ concentration has been proposed to improve test accuracy^16^. These tests consist of an oral load with the corresponding sugar and the monitoring of the altered absorption by measuring the fermentation gases (H_2_ and CH_4_) in exhaled air. An increase in H_2_ and/or CH_4_ levels reflects the intestinal fermentation of non-absorbed sugars. In electrochemistry, the standard hydrogen electrode is an electrode for reference on all half-cell potential reactions and its standard electrode potential is declared to be 0 V at any temperature^17^. In aqueous solutions, the standard hydrogen electrode consists of a platinized Pt electrode and an acidic solution having a unit activity of proton (H^+^) through which H_2_ (gas) supplied at a fugacity of 1.00 bar is passed, ideally in the form of small bubbles so that the electrolyte solution quickly becomes saturated with the gas. The logarithm of the H^+^/H_2_ ratio reveals electrode potential in the solution according to the Nernst equation, while a negative logarithm of H^+^ activity, which refers to the pH scale, is measured by a glass-electrode potential. Similarly, the H_2_ exists at an effective pressure (known as the fugacity) in the gas state, while a hydrogen electrode potential is calculated by pH and the H_2_ effective pressure.

The partial pressures of the separate dissolved gases are designated the same as the partial pressures in the gas state—that is, arterial partial pressure of O_2_ (PaO_2_) and arterial partial pressure of carbon dioxide (PaCO_2_)^18^. Blood gases are mixed in vivo and not equilibrated. Although it is not practical to determine the mixed potential in vivo, the redox environment is considered as the summation of the products of the reduction potential and reducing the capacity of the linked redox couple^19^. O_2_ is an oxidizing agent, and H_2_ is an effective reducer. O_2_ causes a rise of oxidation–reduction potential, and H_2_ causes its reduction in the media of bacterial culture^20^. However, the electrode potential represents the half-cell reaction under certain H_2_ effective pressure against the standard hydrogen electrode (E^0^ = 0)^21^. Therefore, it is practical to evaluate the difference between the two electrode potentials under different H_2_ partial pressure wherever redox environment is common, while pH is measured by the difference between the two electrode potentials in the sample solution and the pH standard buffer solution^22^. Our hypothesis is that changes in the negative logarithm of H_2_ effective pressure in the gas state will indicate variations of electrode potential in the solution, and those between before and after daily activities can be measured using a portable breath H_2_ sensor in healthy Japanese subjects.

## Results

### Oxidation–reduction potential experiments

The H_2_ electrode was based on the redox half-cell reaction: 2H^+^(aq) + 2e^−^ ⇄ H_2_(g). The electrode potential was calculated from the activity of H^+^ and the fugacity of H_2_ using the Nernst equation, as follows:

$$ {\text{E}} = {\text{E}}_{0} - \frac{RT}{{2F}}{\text{ln}}\frac{{f_{{H_{2} }} }}{{\left( {a_{{H^{ + } }} } \right)^{2} }} = - \frac{2.303RT}{{2F}} \times \log \frac{{f_{{H_{2} }} }}{{\left( {a_{{H^{ + } }} } \right)^{2} }}\;\;\left( {\text{V}} \right), $$

where $a_{{H^{ + } }}$ is the activity of H^+^, and $f_{{H_{2} }}$ is the fugacity of H_2_, which, at low pressure, is equal to the ratio of the partial pressure of H_2_ (P_H2_) over the standard pressure ($P^{0} = 1 bar = 10^{5} Pa$). pH is defined as ${\text{pH}} = { } - log\left( {a_{{H^{ + } }} } \right)$. In this study, the pH_2_ scale is defined as

$${\text{pH}}_{2} = - \log \left( {f_{{H_{2} }} } \right) = - {\text{log}}\left( {\frac{{{\text{P}}_{{{\text{H}}2}} }}{{{\text{P}}^{0} }}} \right)$$.

The redox potential can be calculated from pH and pH_2_:

$$ {\text{E}}_{H2} = \frac{2.303RT}{{2F}} \times \left( {pH_{2} - 2pH} \right) \left( V \right) $$

The value of 2.303RT/2F, the Nernst slope, was calculated at 22 °C, where R is the gas constant (R = 8.314 J K^−1^ mol^−1^), T is the temperature (T = 295.15 K), and F is the Faraday constant (F = 9.6485 × 10^4^ C mol^−1^); this equation yields results in mV, as follows:

$$ 2.303{\text{RT}}/2{\text{F}} = \frac{2.303 \times 8.314 \times 295.15}{{2 \times 9.6485 \times 10^{4} }} \times 10^{3} = 29.3. $$

**Figure 1:**
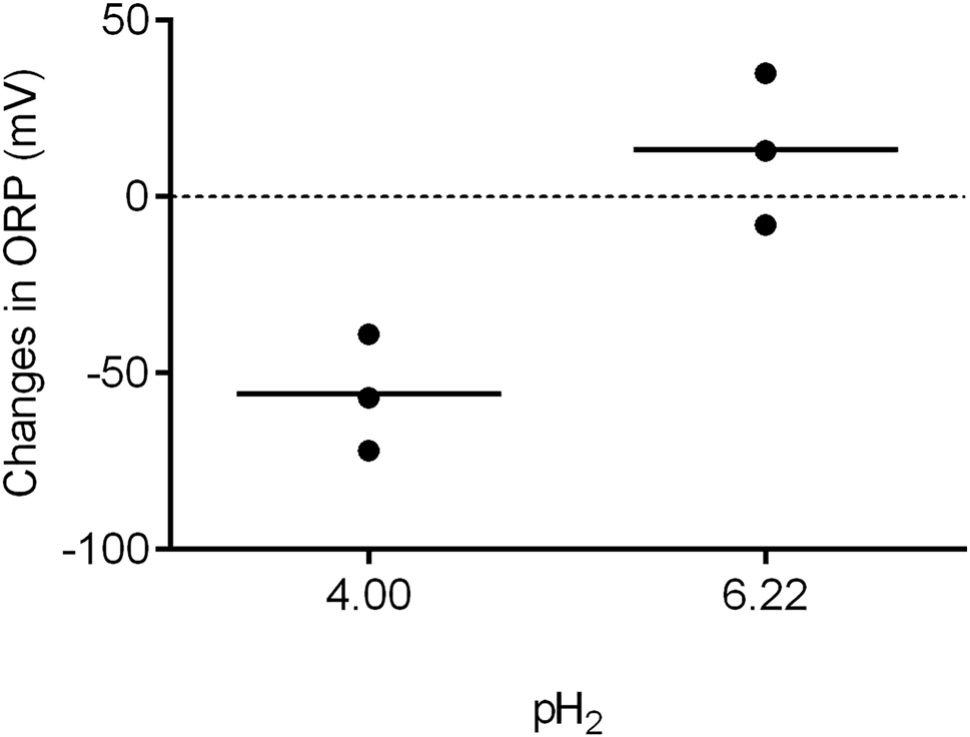
Changes in oxidation–reduction potential (ORP) in phosphate-buffered solution after bubbling air containing 10 Pa of H_2_ (pH_2_ = 4.0, n = 3) and the atmosphere (pH_2_ = 6.22, n = 3*)* (*P* = 0.011). ${\text{pH}}_{2} = - {\text{log}}\left( {\frac{{{\text{P}}_{{{\text{H}}2}} }}{{{\text{P}}^{0} }}} \right)$, $P^{0} = 1\; {\text{bar}} = 10^{5} \;{\text{Pa}}$.

Changes in the oxidation–reduction potential (ORP) were measured after bubbling into a phosphate buffer solution of 100 parts per million (ppm) of standard H_2_ gas and medical air to calculate the slope of ΔORP/($pH_{2} - 2 \times pH$) at room temperature (T = 22 °C). The upper limit of pH_2_ is 6.22, as yielded by 0.06 Pa-H_2_ ($0.6 \times 10^{ - 6}$ bar) in medical air, and the lower calibration point of pH_2_ is 4.0, as yielded by 10 Pa-H_2_ standard gas ($1 \times 10^{ - 4}$ bar). In phosphate buffer solution (pH = 7.1), the ORP changed by − 56.7 ± 17.0 mV after bubbling of the standard H_2_ gas and by a much lower degree (+ 13.3 ± 21.5 mV) after bubbling of the medical air (p = 0.011; Fig. 1).

The pH value after bubbling of the standard H_2_ gas increased to 7.18 ± 0.03 in the phosphate-buffered solution compared to that of the medical air (7.08 ± 0.01, p = 0.0017). The Nernst slope between H_2_ partial pressures of 0.06 Pa and 10 Pa was calculated as

$$ {\text{ Nernst}}\;{\text{ Slope}}\; = \;\frac{{\Delta E_{H2} }}{{\Delta \left( {pH_{2} - 2 \times pH} \right)}} = \frac{{ + 13.3 - \left( { - 56.7} \right)}}{{\left( {6.2 - 2 \times 7.08} \right) - \left( {4.0 - 2 \times 7.18} \right)}} = 29.2 $$

The measured ORP/($pH_{2} - 2 \times pH$) slope agreed with the calculated Nernst slope (2.303RT/2F) under the low partial pressure of H_2_ (0.06–10 Pa). The ORP was proportional to $\left( {pH_{2} - 2 \times pH} \right)$ in the phosphate buffer solution.

### Clinical study

The end-tidal H_2_ is the level of H_2_ released at the end of an exhaled breath. The end-tidal H_2_ levels reflect the adequacy with which H_2_ is carried in the blood back to the lungs and exhaled. The median end-tidal H_2_ partial pressure at baseline was 1.3 Pa, varying within the range of 0.06 Pa (the atmospheric level) to 7.9 Pa (Table 1). Neither the timing of the most recent meal nor the timing of the most recent bowel movement had any influence on end-tidal H_2_ measured at baseline.

**Table 1:** Values of end-tidal H_2_ at baseline and after daily activities in healthy Japanese subjects

Variables (n)	Baseline (Pa)	P^†^	After daily activities (Pa)	P^‡^
All (149)	1.3 (0.5–2.6)		0.9 (0.4–1.9)	0.007
Gender				
Male (51)	1.4 (0.6–2.8)	0.221	0.8 (0.4–1.9)	0.045
Female (98)	1.1 (0.5–2.5)		1.0 (0.4–1.9)	0.062
Last meal				
Breakfast (114)	1.2 (0.5–2.6)	0.197	1.0 (0.4–2.2)	0.240
Overnight fasting (35)	1.6 (0.7–2.9)		0.7 (0.4–1.4)	0.001
Bowel movement				
Yes (74)	1.2 (0.5–2.3)	0.252	0.8 (0.4–1.8)	0.160
No (75)	1.4 (0.6–2.7)		1.0 (0.5–2.0)	0.016

Data are presented as the median values (25th–75th percentile).

^†^Male vs. female subjects, or yes vs. no.

^‡^Baseline vs. after daily activities.

**Figure 2:**
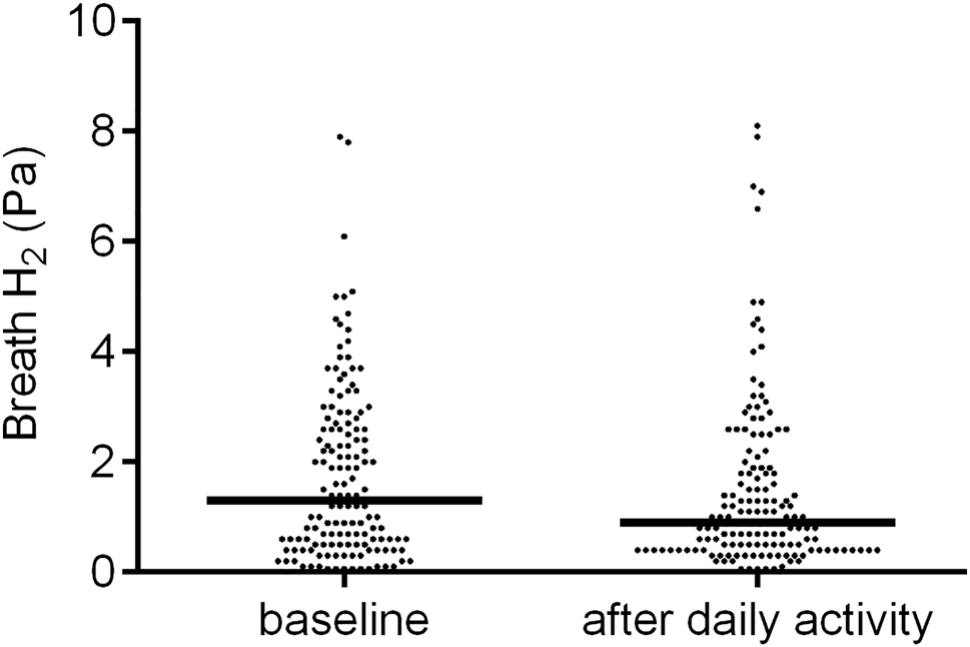
Breath hydrogen (H_2_) partial-pressure measurements at baseline and after daily activities. Breath H_2_ is end-tidal H_2_ partial-pressure (Pa). The median end-tidal H_2_ value of breath after daily activities was lower than the median value recorded at baseline (*P* = 0.007).

The median end-tidal H_2_ after daily activities was 0.9 Pa, which was significantly lower than the median value recorded at baseline (*P* = 0.007, Fig. 2). Of all the subjects, 114 had eaten breakfast within 2 h of the baseline measurement, and no significant difference in H_2_ partial pressure was observed between the values recorded at baseline and those recorded after daily activities. Before the baseline measurement in the morning, 74 subjects had had bowel movements, and no significant difference in H_2_ partial pressure was observed between them.

**Figure 3:**
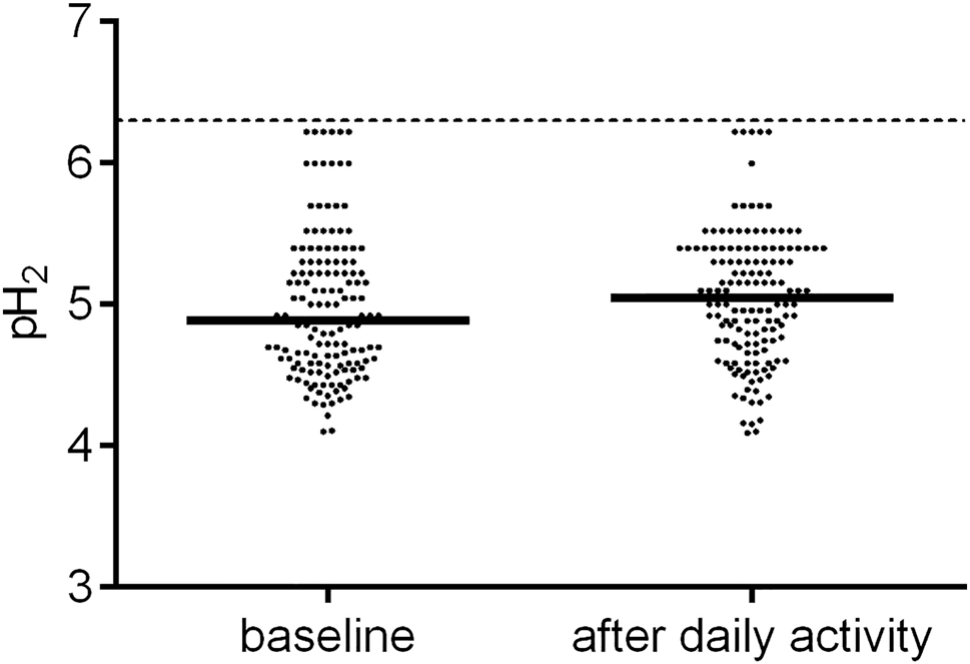
Values of pH_2_ at baseline and after daily activities. The median value of pH_2_ after daily activities was higher than that recorded at baseline (*P* = 0.038).

**Figure 4:**
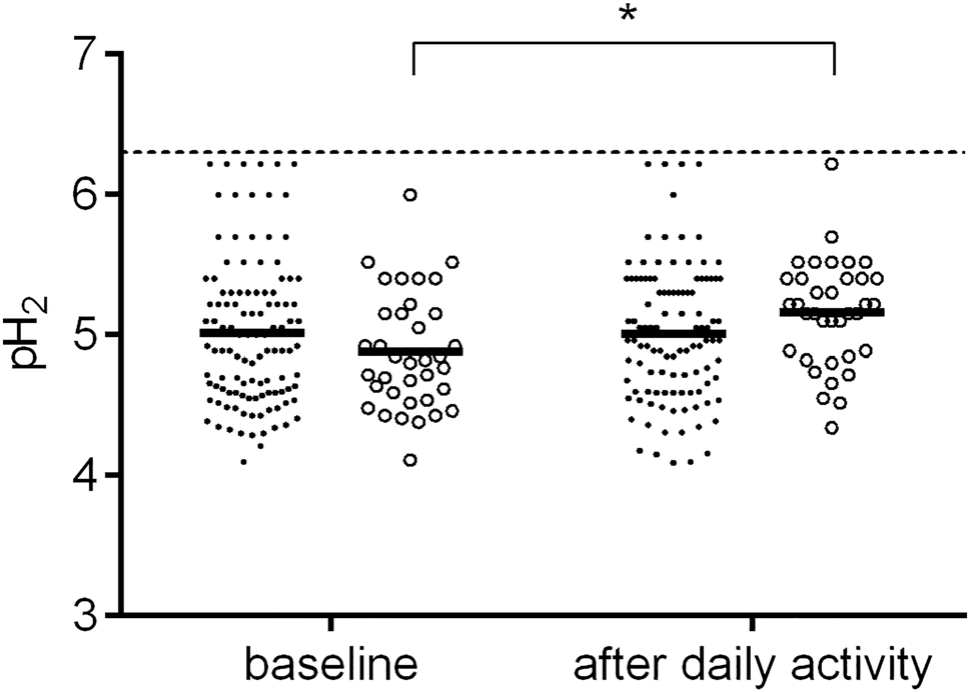
Values of pH_2_ at baseline and after daily activities in the subjects with/without breakfast. The median value of pH_2_ after daily activities was higher than that recorded at baseline in the subjects who had eaten breakfast * (*P* = 0.001). The closed circle [●] indicates the subjects who had eaten breakfast and the open circle [○] indicates the subjects who had not eaten breakfast.

The median pH_2_ value was 4.89 at baseline, varying within the range of 4.10 to 6.22, and it increased to 5.05 after daily activities, varying within the range of 4.06 to 6.22 (*P* = 0.038, Fig. 3, Table 2). A significant difference in the pH_2_ value recorded between baseline and after daily activities was noted in the subjects who had not eaten breakfast (Fig. 4).

**Table 2:** Values of pH_2_ at baseline and after daily activities in healthy Japanese subjects

Variables (n)	Baseline	*P*^†^	After daily activities	*P*^‡^
All (149)	4.89 (4.59–5.30)		5.05 (4.72–5.40)	0.038
Sex				
Male (51)	4.85 (4.55–5.22)	0.221	5.10 (4.72–5.40)	0.018
Female (98)	4.96 (4.60–5.30)		5.02 (4.72–5.40)	0.367
Last meal				
Breakfast (114)	4.92 (4.59–5.30)	0.197	5.00 (4.64–5.40)	0.605
Overnight fasting (35)	4.80 (4.54–5.15)		5.15 (4.85–5.40)	0.001
Bowel movement				
Yes (74)	4.92 (4.64–5.30)	0.252	5.13 (4.74–5.40)	0.488
No (75)	4.85 (4.57–5.22)		5.00 (4.70–5.30)	0.023

Data are presented as median (25th–75th percentile) values.

^†^Male vs. female subjects or yes vs. no.

^‡^Baseline vs. after daily activities.

## Discussion

The ORP of phosphate-buffered solution was changed according to the Nernst equation after air bubbling with and without low concentrations of H_2_ and changes in ORP measured were proportional to $\left( {pH_{2} - 2 \times pH} \right)$. In this study, the end-tidal H_2_ decreased significantly from baseline to after daily activities, and the median pH_2_ at baseline was significantly lower than that after daily activities. However, the Nernst slope is proportional to temperature; thus, the Nernst slope (2.303RT/2F) = 30.8 at 37 °C (310.15 K). Because pH remains within the narrow normal range through acid–base homeostasis, the description of changes in electrode potential sidesteps the need to determine the pH value; $\Delta E \approx 30.8 \times \left[ {pH_{2} \left( {after \;daily\; activities} \right) - pH_{2} \left( {baseline} \right)} \right]$ (mV). After daily activities, pH_2_ was found to increase to 0.15 over baseline in this study, and + 4.6 mV oxidation was estimated. It is suggested that changes in pH_2_ indicate the variation of electrode potential due to alveolar air.

Excretion of H_2_ in breath commonly persists despite an overnight fast. Although elevation of H_2_ concentration above the fasting value after administration of a test sugar is evidence of malabsorption, the significance of the fasting value itself is unknown^23^. Long-term (six months) and daily inhalation of H_2_ reduced the body weight and visceral fat volume compared to the control in rats^24,25^. The decrease in the maximum performance of the skeletal muscle was significant at the beginning of the introduction of 3.1 MPa of H_2_ into the breathing mixture, then returned to the control level at 0.9 MPa during decompression in humans^26^. Excess H_2_ may reduce body weight and muscular performance. However, in young and healthy people, H_2_ inhalation improved running performance and torso strength^27^. In other research, H_2_ reduced delayed-onset muscle soreness after running downhill^28^. These data suggest that little remains known about the proper range of H_2_ through atmospheric, biological, and anthropogenic sources.

Breath H_2_ excretion depends, not only on feeding and fasting patterns, but also on host organ functions^14^. Breath H_2_ excretion is reduced by the consumption of a low-carbohydrate diet during the 24-h period preceding measurement and by fasting during the 12-h period preceding measurement^29^. Resistant starch and dietary fibers have been shown to increase breath H_2_ excretion, while cellulose reduces it^30,31^. When breath H_2_ tests were performed on subjects who consumed a stable diet, some variation in H_2_ was observed^32^. High fasting breath H_2_ was documented in patients with pancreatic exocrine insufficiency and those with small intestinal bacterial overgrowth^33,34^. Low fasting H_2_ concentrations have been reported in patients with congestive heart failure and those with Parkinson’s disease^35,36^. Elderly women show lower baseline and peak H_2_ concentrations than young women^37^. In this study, a meal consuming within 2 h before measurement (breakfast) did not have any influence on the end-tidal H_2_ at baseline.

There was a clear circadian pattern of breath H_2_, high in the morning, decreasing to the nadir by 16:00, increasing again during the night in young women^38,39^. The average profile of breath H_2_ excretion during prolonged exercise was similar to that observed during the control study, in which each participant sat on a chair and lay down^40^. In individuals with baseline H_2_ levels above cutoff (20 ppm), new samples were taken after a 1-h light walk. The decrease in H_2_ levels at 8 ppm and the influence of time of day, before or after 10:00 a.m., may be related^41^. Some of the young female students showed a bimodal pattern of breath H_2_ excretion, high in the morning and decreasing later in the day, then increasing early in the afternoon and later decreasing. This was probably caused by the malabsorption of breakfast^42^. In this study, some of the subjects who ate breakfast showed increased H_2_ excretion at noon. Changes in end-tidal H_2_ after daily activities could be counteracted by consuming a meal 4–6 h before measurement (breakfast). It is suggested that end-tidal H_2_ might follow either a circadian pattern or bimodal pattern.

H_2_ is not directly involved in cellular metabolism, and its transport has not been thoroughly investigated^43^. Some of the H_2_ produced by the microbiome is absorbed into the blood, and dissolved H_2_ diffuses into the alveolar air according to the Bunsen absorption coefficient (1.8 mL of H_2_/100 mL of water at 1 bar) and the ventilation-perfusion ratio (1.35 ± 0.22), which is the ratio of alveolar air (ℓ) per blood (ℓ) in normal-weight individuals aged 25–34 years^44^. As a result, the dissolved H_2_ will be diluted approximately 75 times $\left( {\frac{100}{{1.8}} \times 1.35} \right)$ in the gas phase. The human lung has reciprocating ventilation with large terminal air space (alveoli). The volume of alveolar replaced by new atmospheric air is only one-seventh of the total (350 mL of alveolar ventilation/2300 mL of functional residual capacity)^18^. The slow replacement of alveolar air is of particular importance in preventing sudden changes in the concentration of gases like O_2_ and CO_2_ in the blood. The H_2_ of alveolar gas equilibrates arterial blood during gas exchange. Extracellular fluids and tissues are saturated by H_2_ in arterial blood. H_2_ remains in the tissues until the H_2_ partial pressure in the lungs drops below that of the relevant tissues^45^.

The concentration of dissolved H_2_ at the baseline (1.3 Pa = 1.3 $\times$ 10^−5^ bar) was estimated to be $10.1 \times 10^{ - 9}$ M (10.1 nM) using Henry’s law constants, as follows: H^cp^ = $7.8 \times 10^{ - 4} \left( {\frac{{{\text{mol}}}}{{{\text{L}}\;{\text{bar}}}}} \right)$^46^. The concentration of dissolved H_2_ was similar to the arterial H^+^ concentration at pH = 7.4, which is converted as $10^{ - 7.4} = 39 \times 10^{ - 9}$ M (39 nM). In this study, 10 mM of phosphate-buffered solution $\left( {H_{2} PO_{4}^{ - } \rightleftarrows H^{ + } + HPO_{4}^{2 - } } \right)$ was alkalizing (pH = 7.18 after air-bubbling with H_2_ and pH = 7.08 after air bubbling without H_2_). In the 1960s, Japan’s health authorities endorsed the alkaline water ionizer as a medical device capable of producing both alkaline and acidic water. Alkaline ionized water and electrolyzed reduced water have alkaline pHs and high concentrations of dissolved H_2_. Drinking dissolved H_2_ caused serum alkalinization in the healthy human volunteers^47^. Dissolved H_2_ does not directly affect the pH of a solution $\left( {2H^{ + } + 2e^{ - } \rightleftarrows H_{2} } \right)$. However, dissolved H_2_ in the solution may influence pH measurement because uncertainty budget for the standard potential of the Ag|AgCl electrode includes ${\text{log}}\left( {\frac{{{\text{P}}_{{{\text{H}}2}} }}{{{\text{P}}^{0} }}} \right)$^22^.

Under normobaric conditions, pH_2_ ranges from 0 (1 bar of H_2_ gas) to 6.2 (0.6 ppm of H_2_ in the air). In this study, the median pH_2_ at baseline was 4.89 and the 25th–75th percentile range of pH_2_ was 4.59–5.30, which is broader than the normal pH range of 0.1. The electrode potential of a H^+^/H_2_ pair can be calculated using pH_2_ and pH as $E \approx 30.8 \times \left( {pH_{2} - 2 \times pH} \right)$(mV) at 37 °C. When local blood flow is measured in vivo, the current between a calomel half-cell and a platinum electrode in the tissue is proportional to the H_2_ level^48^. The pH_2_ scale could serve as a measure of how reduced or oxidized water is wherever redox environment is common, given that pH is a measure of how acidic or basic water is^49^.

To gain a more differentiated understanding of cellular redox biology, quantitative, redox-couple specific, in vivo measurements are necessary^50^. Plasma cysteine/cystine and GSH/GSSG are oxidized at different rates as a function of age^51^. Those redox states undergo diurnal variation, and the mean differences between maximal and minimal redox state values for cysteine/cystine and GSH/GSSG were 6 and 4.5 mV, respectively, in 63 samples of healthy human plasma^52^. In the context of redox-sensitive proteins, a 6-mV difference is equivalent to a 1.6-fold increase in the ratio of dithiol to disulfide forms. However, in vitro studies have shown that variation in cysteine/cystine redox over the range found in vivo affects signaling pathways that control cell proliferation and oxidant-induced apoptosis^53^. Moreover, 1.3–5.0% H_2_ regulates various signal transduction pathways and the expression of many genes in human monocytic cell line^54^. Hepatic oxidoreduction-related genes are upregulated by the administration of 0.7 mM-H_2_ drinking water in rats^55^. Recently, it has been reported that all collaborating metabolic organs can be regulated through changes in circulating redox metabolites regardless of whether the change was initiated exogenously or by a single organ^56^. In this study, the variation of electrode potential was estimated to be 4.6 mV between before and after daily activities. It is rational to measure pH_2_ as an electrode potential indicator that predicts organ functions and signaling pathway in vivo.

We acknowledge that while this investigation offers insight into a novel description of electrode potential variation in humans, there are limitations to this technology that must be overcome before it can be validated. H_2_ is not a metabolite of human cells but instead an environmental factor that acts through H_2_-producing microbiomes. Intracellular electrode potential depends on the oxidized/reduced forms of metabolites in each intracellular compartment. The extracellular electrode potential affects the intracellular electrode potential, and the electrode potential depends on environmental factors such as O_2_ and H_2_. The electrode potential is heavily influenced by 21% O_2_ in the atmosphere and by less than 20 ppm of H_2_ in the alveolar air under normoxia. This investigation provides the use of real-time, non-invasive means of estimating the variation of electrode potentials in healthy humans.

## Materials and methods

### Oxidation–reduction potential experiments

The effect of H_2_ at low partial pressure was evaluated using the ORP of a phosphate buffer solution (10 mM at pH = 7.1; Fujifilm Wako Pure Chemical Corp., Osaka, Japan). For a mixture of gases at low pressure, the activity is equal to the ratio of the partial pressure of the gas over the standard pressure. The standard H_2_ gas contains 100 ppm of H_2_ ($1 \times 10^{ - 4} \;{\text{bar}})$ in medical air, and the air contains 0.6 ppm of H_2_ ($0.6 \times 10^{ - 6} \;{\text{bar}}$). The phosphate-buffered solution was not aerated before the experiment, although tap water has a positive ORP ranging from + 200 to + 400 mV due to dissolved O_2_. The air was bubbled in a phosphate-buffered solution at a rate of 0.5 L/min using a humidifier water bottle for 60 min at 22 °C. The air was supplied from the integrated piping system at the TMG Asaka Medical Center (Air Water Inc., Osaka, Japan), and standard H_2_ gas for calibration (100 ppm of H_2_ in medical air; Air Water Inc., Osaka, Japan) was supplied from the cylinder with a pressure regulator. The ORP and pH were measured before and after the air bubbling using an ORP/pH meter (pH6600 and ORP6600S; Custom, Tokyo, Japan).

### Clinical study

#### Study design

This was a non-randomized, non-blinded, prospective observational study performed to evaluate the variation of electrode potential using end-tidal H_2_ levels in healthy Japanese subjects. This study’s protocol was approved by the ethical review board of TMG Asaka Medical Center. The trial (UMIN000014696) was registered in the University Hospital Medical Information Network in Japan in May 2018. All participants provided written informed consent before enrollment. The study was performed between September 2018 and December 2018 at the TMG Asaka Medical Center in Saitama.

#### Participants

We enrolled 160 healthy, non-obese Japanese subjects (BMI < 30). We then excluded 11 smokers. Of the remaining 149 subjects, 98 were women and 51 were men, with an average age of 29.8 ± 9.0 years. All methods were performed in accordance with the relevant guidelines and regulations^28^. In this study, H_2_ concentration was measured using a portable handheld H_2_ breath test apparatus (Gastrolyser; Bedfont Scientific Ltd., Kent, UK) after 15 s of breath-holding^57,58^. The time point at which the concentration of H_2_ in the breath equilibrated or flattened to that in the blood was 15–20 s. Then, subjects were asked to exhale slowly but gently into the mouthpiece, aiming to empty their lungs completely for accurate breath analysis with end-tidal samples^59^. Measurements taken at 8:00 a.m. served as the baseline. The subjects were then allowed to start their daily activities, which included in-hospital desk and laboratory work as well as physical therapy. Measurements were taken again after daily activities at noon but before lunch to prevent eating and drinking from influencing the breath test results. Calibration was performed using standard H_2_ gas (100 ppm of H_2_ gas in medical air, 0.5 L/min of flow) as 100 ppm, and the atmospheric pressure was set to 0 points. The end-tidal H_2_ partial pressure was calculated as follows: $P = \left( {H_{2} \; concentration} \right) \times 100\;{\text{kPa}}$ or 0.06 Pa at 0 points of H_2_ concentration. End-tidal hydrogen levels were measured by a hand-held electrochemical H_2_ sensor. Electrochemical gas sensors have become popular due to their linearity of output. In addition, once the correction is performed with a known concentration of the target gas, it provides excellent repeatability and accuracy during measurements. Technological advances over the past few decades have enabled electrochemical gas sensors to show very good selectivity for specific gas types. In addition, although selectivity for the target gas has been greatly improved, the cross-sensitivity to other gases is still not zero, increasing the possibility of interference and misdirection during measurements. The detection range of the H_2_ sensor is 0–500 ppm. The accuracy is ± 10% and CO cross interference is less than 1%. Accurate measurement of H_2_ in ppm in expiratory air reveals intolerance and/or malabsorption of carbohydrates; or bacterial over growth.

Before testing, all subjects answered a questionnaire covering meals, exercise habits, and bowel movements.

#### Statistical analyses

All statistical analyses were performed using IBM SPSS Statistics, version 25 (IBM Corp., Armonk, NY, USA). Values of pH_2_ are presented as medians and interquartile ranges. The univariate analysis was generally based on non-parametric methods (Wilcoxon or Mann–Whitney *U*). *P* < 0.05 was considered statistically significant.

## Data Availability

The datasets used and/or analyzed during the current study are available from the corresponding author upon reasonable request.
